# P05 Blast transformation of essential thrombocythemia presenting as hip monoarthritis

**DOI:** 10.1093/rap/rkab068.004

**Published:** 2021-10-19

**Authors:** Sarah Loughrey, Claire Benson, Claire Arnold

**Affiliations:** 1 Rhuematology, Musgrave Park Hospital, Belfast, United Kingdom; 2 Haematology, Belfast City Hospital, Belfast, United Kingdom

## Abstract

**Case report - Introduction:**

We present a rare case of a patient with a culture-negative mono arthritis. Our patient had a history of Essential Thrombocythemia (ET), and his mono arthritis initially responded to antibiotics. He had a fluctuating clinical course, and a multi-disciplinary approach with rheumatology, orthopaedics, haematology and radiology was needed. After multiple MRIs and two bone biopsies, he was diagnosed with acute myeloid leukaemia (AML) with blast transformation of his myeloproliferative neoplasm, essential thrombocythemia.

This case highlights the need to consider the differentials of mono arthritis despite initial response to treatment, and the value of a multi-disciplinary approach.

**Case report - Case description:**

A 59-year-old male was referred to rheumatology with new right hip pain, and high inflammatory markers. He was negative for infection, trauma, seronegative or crystal arthritis. Past history of ET diagnosed in 2011 with high-risk genetics. At haematology review 3 months prior he had new pancytopenia with deranged LFTs. A bone marrow biopsy (BMBx) showed post-ET myelofibrosis with no circulating blasts. His hydroxycarbamide and aspirin were stopped and blood counts recovered without other intervention.

He was afebrile and had a right hip effusion. The hip aspirate revealed no growth after 48 hours and negative crystals.  Bloods: Hb 101 WCC 5.1 Neutrophils 3.8 Platelets 318 CRP 276 ESR 72 Urate normal. MRI showed a right hip joint effusion, with extensive soft tissue oedema likely secondary to infection. He was commenced on IV Flucloxacillin, clinically improved and was discharged to complete antibiotics. At review in 2 weeks he had worsening hip pain, night sweats, raised CRP, and was anaemic with normal WCC and platelets. A repeat MRI showed evolving abscess formation within the acetabular roof.

After discussion with haematology and orthopaedics, the clinical impression was septic arthritis as he was responding to treatment, his blood film was normal and recent BMBx showed no transformation. The plan was to continue with antibiotics and to perform a bone biopsy. Bone biopsy showed necrotic debris secondary to the underlying osteomyelitis. Due to fluctuating course a repeat MRI was performed showing a larger hip effusion with appearances suspicious for malignancy. A repeat bone biopsy showed post ET fibrosis with blasts and large tumour cells. A blood film showed peripheral blasts circulating. The unifying diagnosis was AML with blast transformation of myeloproliferative neoplasm. Our patient was refractory to chemotherapy. He was treated with palliative radiotherapy and died with palliative care input.

**Case report - Discussion:**

Septic arthritis typically affects one joint and mimics include abscess, AVN, cellulitis, crystal, malignancy, reactive and inflammatory arthritis, and transient synovitis. Synovial fluid analysis is the gold standard for the diagnosis and will demonstrate growth in approximately 80% of all cases of non-gonococcal septic arthritis. The remaining 20% are ‘culture negative’ and may demonstrate no growth for a variety of reasons. 14% of patients with ‘culture negative’ septic arthritis will have positive blood cultures and newer diagnostic tools such as PCR testing of synovial fluid increases the likelihood of correctly identifying an organism. Our patient initially responded to treatment for septic arthritis; however, the atypical clinical course and history of ET prompted further investigations for differentials of ‘seronegative arthritis.’

ET is a myeloproliferative neoplasm with a prevalence of 40/100000. It is usually indolent and recognised genes are JAK2, CALR and MPL. Complications include vascular events such as thrombosis and haemorrhage. More serious complications of transformation to myelodysplasia, AML or myelofibrosis are rare with an incidence of 1—5% and median time to transformation is between 84 and138 months.

Rheumatic manifestations of leukaemia include leukaemia arthritis. This is uncommon and develops due to infiltration of synovial membrane by leukaemia cells. Synovial fluid or synovial biopsy can be analysed for blasts and flow cytometry. The reported incidence is 12% in children, and only case reports in adults.

The blast infiltrative phase of leukaemia usually occurs in the bone marrow, or blood. Previous case reports describe extra medullary blasts present in lymphoid tissues, and here we report an extra medullary blast sarcoma. Transformation of ET to AML is typically very difficult to manage, with a poor prognosis. Treatment options include chemotherapy, stem cell transplants and the JAK inhibitor ruxolitinib.

**Case report - Key learning points:**

Differentials for a mono arthritis are wide and this is a rare case of hip mono arthritis as the clinical presentation of transformation of an ET to AML.

Septic arthritis is one of the most important diagnoses to rule out in a patient with a mono arthritis and high inflammatory markers. Up to 20% of septic arthritis can be ‘culture negative.’ Peripheral blood cultures and PCR testing of synovial fluid can be used to enhance the identification of organisms.

Although our index of suspicion was high, our patient was not displaying typical manifestations of transformation to AML. The development of an acute leukaemia can be insidious, and case reports have described infiltration of bone marrow simultaneously or months following an extra medullary blast crisis. This case highlights the importance of further, and if necessary repeated, investigations in a patient with an atypical history or clinical course. Malignancy as a differential for mono arthritis is important to consider as mis diagnosis could delay treatment and prognosis is very different. Both bone biopsies yielded abnormal bone; however, it was the second biopsy that showed circulating blasts. In our case the bone biopsies were performed without radiological guidance, and studies have shown that CT guided biopsies have a higher yield. In future, consideration could be given to utilising radiologically guided biopsy.

A multi-disciplinary approach between a number of specialities is often needed in complex cases. This case highlights the value of this with collaboration with our haematology, orthopaedic, radiology and pathology colleagues.

